# Hematuria as a Sign of Kidney Stone Disease Evaluated Using Computed Tomography: A Review

**DOI:** 10.7759/cureus.38064

**Published:** 2023-04-24

**Authors:** Vadlamudi Nagendra, Rajasbala Dhande, Gaurav Mishra, Nidhi G Reddy, Harshith Gowda

**Affiliations:** 1 Radiodiagnosis, Jawaharlal Nehru Medical College, Datta Meghe Institute of Higher Education and Research, Wardha, IND

**Keywords:** ct, ultrasound, renal calculi, hematuria, computed tomography

## Abstract

Kidney stone is a common cause of acute pain in the abdomen in patients presenting to casualty. Being present in roughly 12% of the world's population makes it the most prevalent pathology of the urinary system. The ureters, kidneys, and bladder frequently develop calculi, resulting in hematuria. The most effective imaging technique for evaluating calculi is unenhanced helical computed tomography. The population, intervention, control, and outcomes (PICO)-formatted question was used to generate methodological medical subject heading (MeSH) phrases, which increased the search strategy's sensitivity in finding research. Some of these names ("hematuria") included "renal calculi" (MeSH) and "cone-beam computed tomography" (MeSH). Studies that satisfied these requirements were subjected to critical evaluation. The merits of the listed studies were evaluated using a unique quality assessment scale. The most accurate imaging diagnostic test for people with hematuria is multidetector computed tomography. If a patient over 40 presents with microscopic hematuria, a non-contrast computed tomography or ultrasound study should be performed, and if gross hematuria is observed, cystoscopy should be added. Pre- and post-contrast computed tomography scans and cystoscopy should be carried out on elderly patients.

## Introduction and background

Kidney stone is a common cause of acute pain in the abdomen in patients presenting to casualty. Being present in roughly 12% of the world's population makes it the most prevalent pathology of the urinary system [1,2]. With a peak age range of the second to the fourth decade, males are more likely than females to experience it. If no prophylaxis is given, the patient has a significant secondary recurrence rate of 10% to 23% per year, 50% in 5-10 years, and 75% in 20 years [3]. Kidney stone disease is a significant pathology to discuss because both developing and developed countries are experiencing worrying increases in frequency. Urinary stones are predicted to affect 12% of Indians, and in 50% of cases, kidney impairment follows [1]. Recurring kidney stones cannot currently be prevented or treated effectively. Lack of adequate fluid intake results in decreased urine production and a high concentration of salts that can form renal calculi. More than 75% of all stones are calcium, the most frequent form. Renal colicky pain which manifests as intense crying that may seem more like screaming or an expression of pain radiating to the flank, nausea, vomiting, hematuria (blood in the urine), chills and rigors, oliguria or anuria, and dysuria are the main symptoms that present [4].

The presence of any number of red blood cells (RBCs) per high-power field (HPF) during the microscopic analysis of the urine is defined as microscopic hematuria. In contrast, gross hematuria is described as passing visible blood in urine, confirmed with microscopic examination. Hematuria may be brought on by calculi, neoplasms, infections, trauma, medication poisoning, coagulopathy, and varices, among other things. Sometimes, the reason might be determined by a patient's clinical history of strenuous exercise or a recent catheterization. The ureters, kidneys, and bladder frequently develop calculi, resulting in hematuria [5].

The most effective imaging technique for evaluating calculi is unenhanced helical computed tomography. Although ultrasound helps find renal calculi, it is sometimes challenging to see ureteral calculi. Urinary calculi can be found with conventional radiography, although this method lacks the sensitivity of unenhanced computed tomography [6-8]. To analyze the burden and severity of the disease as well as the sensitivity of computed tomography in identifying calculi, the objective of this systematic review article is to establish the incidence of kidney stone disease in patients with hematuria using radiological findings on computed tomography assisting the radiologist and doctor in the administration of efficient prevention and therapy.

## Review

Materials and methods

The National Institute for Health Research PROSPERO International Prospective Register of Systematic Reviews received the current systematic review and recorded it there. We looked through the electronic MEDLINE, Embase, Cochrane, and PubMed databases. Additionally, each relevant article's and book's bibliography was thoroughly searched. Based on the inclusion and exclusion criteria, the pertinent articles were chosen by two reviewers separately. The two reviewers debated any challenges until they arrived at a consensus.

Methodological medical subject heading (MeSH) phrases were created using the PICO-formatted question to increase the search strategy's sensitivity in locating research. "Renal calculi" (MeSH) and "cone-beam computed tomography" (MeSH) were some of these names ("hematuria" (MeSH)) included. Studies that satisfied these requirements were subjected to critical evaluation. Using a unique quality assessment scale that has been proposed, the merits of the mentioned studies were evaluated.

Eligibility criteria and study selection

Until 2021, studies on renal calculi, hematuria, and computed tomography that were peer-reviewed and published in English-language journals were included. Included studies had outcomes that were defined in terms of radiographic data. Exclusion criteria included case reports, case series, cross-sectional studies, animal or laboratory research, reviews, abstracts, articles with insufficient data, and patients with any lesions. For further research, the references of the chosen papers were also examined. Any research that didn't fit the requirements was excluded.

Data extraction and synthesis

Three steps were taken in the selection of the studies. Examination of all book titles was done, and suitable studies were selected as per the inclusion and exclusion criteria. Abstracts for each of the selected titles were acquired, examined, and relevant abstracts were selected using the criteria. The full texts of all the chosen abstracts were retrieved, examined, and the selected articles were chosen while keeping in mind the selection criteria. Finally, seven articles were selected for the investigation.

The data extraction forms were used to extract the data (Table 1). Authors, study year, study design, patient count, gender, mean age, radiological investigations - ultrasound, computed tomograms of patients having renal calculi, hematuria, with or without other renal disorders and its intervention data - were noted. If patients were monitored, the length of the monitoring and the presence or absence of recurrence were noted for each article.

**Table 1:** Data extraction and synthesis

Initial search	190
Duplicates and non-relevant	81
Case reports and series	13
Reviews	61
Abstract	14
Language other than English	14

Results

**Figure 1:**
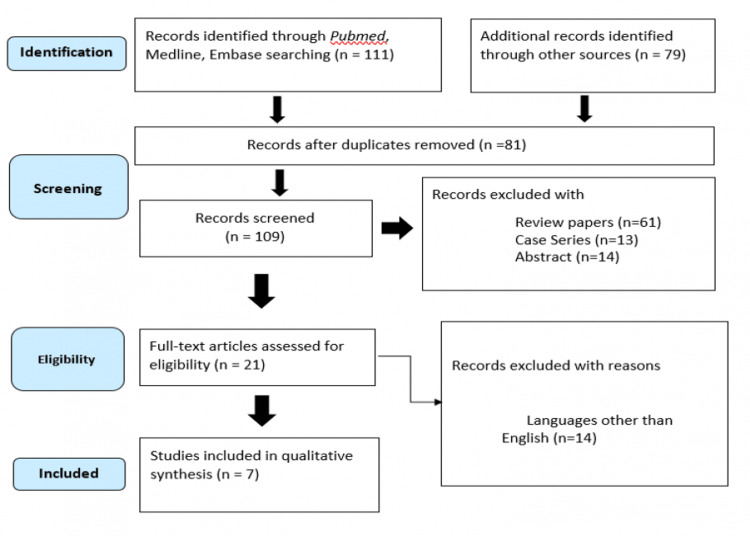
PRISMA flow chart

A total of 190 articles were obtained during the initial search. A total of seven studies were considered for analysis out of 190 articles that were found in the database after duplicates were removed and eligibility criteria had been applied (Figure 1).

The study's quality was evaluated using the Cochrane collaboration method for assessing the risk of bias in randomized controlled trials (RCTs) (Table 2). The Newcastle-Ottawa Quality Assessment Form for cohort studies, the Critical Appraisal Skills Programme, the Cochrane Collaboration's tool for assessing the risk of bias, the Oxford Systematic Review Appraisal Sheet, and the Grading of Recommendations Assessment, Development, and Evaluation (GRADE) system for grading evidence were all used to ensure the accuracy of this data analysis in this systematic review [9-12]. Narrative synthesis has been provided for the findings obtained from the studies. The extracted data has been displayed in tabular format (Table 3).

**Table 2:** Assessment of quality of studies included in a present systematic review

Authors name	Selection bias (random sequence generation)	Allocation concealment	Reporting bias	Others	Performance bias (blinding participants and personnel)	Blinding outcome	Attrition bias
Safriel Y et al. in 2003 [13]	Low risk	Low risk	Low risk	Low risk	Low risk	Unclear	High risk
Lang EK et al. in 2003 [8]	Low risk	Low risk	Low risk	Low risk	Low risk	Low risk	Low risk
Galley RE et al. in 2008 [14]	Low risk	Low risk	Low risk	Low risk	Low risk	Low risk	Low risk
Jin DH et al. in 2010 [7]	Low risk	Unclear	Low risk	Low risk	Low risk	Unclear	Low risk
Carter MR et al. in 2011 [15]	Low risk	Low risk	Low risk	Low risk	Unclear	Unclear	Low risk
Mishriki SF et al. in 2012 [16]	Low risk	Low risk	Low risk	Low risk	Low risk	Low risk	Low risk
Lai WS et al. in 2016 [5]	Low risk	Low risk	Unclear	Low risk	Low risk	Unclear	Low risk

**Table 3:** Extracted data CT, computed tomography; CTU, computed tomographic urography

S. no.	Author and year	Country	Intervention	Outcomes
1	Safriel Y et al. in 2003 [13]	U.S.A.	Abdomen CT	The finding of urinary stones without hematuria does not imply obstruction.
2	Lang EK et al. in 2003 [8]	Los Angeles, U.S.A.	Multiphasic helical CT	Helical CT makes it possible for the diagnosis of early inflammatory disease, small masses and neoplastic lesions, and vascular abnormalities
3	Galley RE et al. in 2008 [14]	U.S.A.	CT scan	A pre- and post-contrast computed tomography and cystoscopy should be performed in older patients.
4	Jin DH et al. in 2010 [7]	California, U.S.A.	CT scan	Multidetector CT scanning parameters should be tailored to minimize radiation exposure to the patients while helping detect clinically significant renal stones.
5	Carter MR et al. in 2011 [15]	U.S.A.	Non-contrast CT	CT demonstrated some periureteral stranding and mild hydroureter but no evidence of a stone.
6	Mishriki SF et al. in 2012 [16]	UK	Renal tract ultrasound/excretory urography/contrast-enhanced computer tomography urogram/flexible cystoscopy/urine cytology	As 11.6% of patients with recurrent visible hematuria will have a malignant pathology after initial negative findings, it is imperative that they should have full standard investigations repeated
7	Lai WS et al. in 2016 [5]	Birmingham, UK	Computed tomography urogram (CTU)	CTU can lead to expensive and invasive testing and treatment

Helical computed tomography identifies early inflammatory illness, tiny masses and neoplastic lesions, and vascular anomalies, according to Lang EK et al. in 2003 [8]. Galley RE et al. in 2008 demonstrated that older individuals should get a pre- and post-contrast computed tomography scan and a cystoscopy [14]. Multidetector computed tomography scanning parameters should be adjusted to reduce radiation exposure to patients while assisting in the detection of clinically significant kidney stones, according to Jin DH et al. in 2010 [7]. In 2011, Carter MR et al. reported that computed tomography showed modest periureteral stranding and a mild hydroureter but no sign of a stone [15]. After initial negative findings, Mishriki SF et al. in 2012 concluded that 11.6% of patients with recurrent vitreous hemorrhage (VH) would have a malignant pathology; therefore, they must undergo thorough routine investigation once more [16]. In 2016, Lai WS et al. showed how computed tomography urography could produce pricy and intrusive testing and therapy [5].

Discussion

The sensitivity of computed tomography in the definitive diagnosis of urinary calculi has been demonstrated in numerous earlier research [17-21]. The use of computed tomography in assessing acute flank pain is rising due to its ability to visualize even small renal stones, their number, location, size, and Hounsfield unit (HU) value. Computed tomography also helps in evaluating complications (hydronephrosis, spontaneous rupture of renal pelvis, pyelonephritis, impaired renal function, and rare incidence of renal malignancy) of renal stone and rules out other causes of acute flank pain. In this work, we demonstrate the unreliability of yet another indirect test for urinary calculi, which was previously believed to be intimately tied to urine calculi. By doing this, we again emphasize the crucial role computed tomography should play in assessing urinary calculi. Not only do we demonstrate that urinary tract calculi can develop in the absence of hematuria, but we also demonstrate that many patients did not have calculi even when hematuria was present.

The relationship between urinary stones and the presence of hematuria has generated significant debate in the literature. A study on 122 patients found that 95.4% had microscopic hematuria, whereas a study on 51 patients with stone disease found that 100% had microscopic hematuria [22,23]. The fact that these studies used intravenous urogram (IVU) and plain radiography to identify stone disease was a significant weakness. Tradition held that the trauma the stone imparted to the urinary tract was what caused hematuria when there were stones present. The cause of cases where hematuria was not found was believed to be the stone completely obstructing the collecting system.

Despite ongoing improvements in multidetector computed tomography technology, patients continue to get significant radiation doses. Ways to reduce radiation exposure in computed tomographic scans are following ALARA (as low as reasonably achievable) principle, using the most dose-efficient technique by optimizing technical aspects, utilizing automatic exposure control settings or manual technique charts, and better data processing and image reconstruction [24]. According to estimates, one in 1000 people may be at risk for acquiring a deadly malignancy in their life after undergoing a single abdomen and pelvic non-contrast computed tomography scan [25]. Patients exposed to radiation at a young age have an increased risk of cancer (one in 550 for a one-year-old having a single abdominal computed tomography scan) [26]. Many doctors are still unaware of the elevated risk associated with computed tomography imaging, even though the risk of radiation exposure for secondary cancer (thyroid cancer, leukemia, and non-Hodgkin's lymphoma) has been discussed periodically [27,28]. Infarctions appearing wedge-shaped with expanding thin cortical rims, showing reperfusion by capsular collaterals, were seen on multiphasic helical computed tomography. The absence of parenchymal striation and intensification of the rim stain during the venous phase are reliable indicators so that the condition can be distinguished from segmental pyelonephritis [29]. Small segmental renal infarcts rarely show signs on IVU because collateral reperfusion of the damaged parenchyma keeps contrast excretion at a normal level and results in normal, albeit somewhat delayed, enhancement of parenchyma. While vascular phase helical computed tomography amply illustrates such anatomic correlations, IVU generates no criteria for detecting an aberrant position of the renal vein [30].

Limitations

The majority of the included research had issues with accurate demographic information, data accessibility, and thorough methodology.

## Conclusions

The most accurate diagnostic investigation for people with hematuria is multidetector computed tomography. If a patient over 40 presents with microscopic hematuria, a non-contrast computed tomography or ultrasound study should be performed, and if gross hematuria is observed, a cystoscopy should be done. Pre- and post-contrast computed tomography scans, as well as a cystoscopy, should be carried out on elderly patients. To avoid ionizing radiation, ultrasound is the first line imaging modality in examining renal stones and their consequences in all patients, including pregnant women. Computed tomography is indicated if the ultrasound examination is inconclusive. The American Urological Association and American College of Radiology consider multidetector computed tomography as the gold-standard technique to asses acute flank pain in patients when there is a clinical suspicion of renal calculi. Non-contrast computed tomography is the most accurate test for renal calculi due to its high sensitivity and specificity in evaluating the number, size, location, morphology of calculus, and its ability to evaluate other renal pathologies. Methods to reduce computed tomographic radiation exposure should be employed in all patients.
